# Optimising registrar research supervision: Quality, conflict management and best practices

**DOI:** 10.4102/jcmsa.v3i1.167

**Published:** 2025-02-12

**Authors:** Adetayo E. Obasa, Ts’epo Motsohi, Marilize Burger, Jeanne Lübbe, Lizelle van Wyk, Jacques du Toit

**Affiliations:** 1 Registrar Research Support Office, Research and Internationalisation Development and Support, Faculty of Medicine and Health Sciences, Stellenbosch University, Cape Town, South Africa; 2 Department of Family and Emergency Medicine, Faculty of Medicine and Health Sciences, Stellenbosch University, Cape Town, South Africa; 3 Division of Orthopaedic Surgery, Faculty of Medicine and Health Sciences, Stellenbosch University, Cape Town, South Africa; 4 Department of Paediatrics and Child Health, Faculty of Medicine and Health Sciences, Stellenbosch University, Cape Town, South Africa

**Keywords:** clinical supervision training, clinician, conflict management, Master of Medicine, registrar, supervisor

## Abstract

Completing a Master of Medicine (MMed) research assignment involves both financial and time commitments. In addition, it requires considerable collaboration among supervisors, registrars, research coordinators, assistants, nurses, and patients. Substantial professional effort is necessary for developing the research concept, reviewing relevant literature, formulating a research question, and designing an appropriate methodology. From this perspective, we reflect on a clinical research supervision workshop that focuses on establishing robust supervision and conflict management practices. The workshop aimed to enhance supervision capacity for MMed research, guiding students in scientific rigour and methodology. By addressing this need, we hope to improve research quality across South African faculties and develop a new generation of skilled clinician-researchers. Clinicians as supervisors, play a pivotal role in guiding students through their academic and professional journeys. Best practices include fostering open communication, setting clear expectations, and providing consistent feedback. Institutional support is crucial, offering resources and training to enhance supervisory skills.

## Introduction

In November 2010, the Subcommittee for Postgraduate Education and Training (Medical) under the Health Professions Council of South Africa (HPCSA) implemented new regulations requiring the completion of a research component as a condition for specialist registration. These regulations became mandatory for all registrars commencing their training on or after 01 January 2011.^1^

Clinical research is integral to advancing healthcare, and incorporating it into specialist training requires careful consideration. For research outcomes to be meaningful, investigators must identify research gaps or questions in the field and design robust methodologies to answer these questions and potentially address the gap. This endeavour requires that clinical research supervisors not only possess strong research skills but also be adept in guiding and mentoring their trainees.^2^ While much attention is often placed on supervisors’ research capabilities, effective supervision also depends on ‘soft’ skills – such as mentorship, communication, and the ability to manage registrars’ dual responsibilities of clinical duties and research.^3^ In some instances, the ability to provide supportive supervision can be the deciding factor as to whether registrars complete their research assignments.

Aside from the time constraint issues, one of the common challenges registrars face is prioritising their clinical duties and Colleges of Medicine of South Africa (CMSA) examination requirements over their research, which often leads to delays in completing the research component necessary for specialist registration.^4^ These delays in registration as a specialist, in turn, have ripple effects on the broader healthcare service in South Africa. However, with effective mentorship, a structured support system, and tailored guidance, these challenges can be mitigated.

From this perspective, colleagues at the Faculty of Medicine and Health Sciences (FMHS) at Stellenbosch University reflect on a 2-half day workshop designed to address these challenges and build clinical research supervision capacity. In the following sections, we will discuss the day-1 events (part 1 of 2) which include: defining quality in research assignments, enhancing Master of Medicine (MMed) supervision, conflict management, and best practices.

## Defining an ideal Master of Medicine

An ideal MMed research project gives a specialist clinician basic skills for research that directly benefits their medical speciality. In a technologically fast-changing clinical landscape, it equips the clinician with tools to practise evidence-based medicine in a future career which may be outside of academic medicine. Unlike a PhD programme, which entails academic research over a 3–4 year period, the MMed is a shorter programme, usually completed in 4 years with some surgical disciplines taking 5 years in tandem with clinical responsibilities. A practical approach towards the MMed research assignment involves developing research skills while simultaneously generating generalisable knowledge that can address specific clinical issues, especially in sub-Saharan African healthcare like human immunodeficiency virus-1 (HIV-1) and acquired immunodeficiency syndrome (AIDS), trauma, advanced disease management, and cost-effective healthcare delivery.

At this workshop, we posed the question: What is a good MMed research topic? The discussion that followed included that an MMed should be practical, feasible to complete within the programme’s time limits, and aligned with evidence-based medicine to increase applicability at the bedside. Clinician supervisors play a key role, guiding students on topic choice and methods to make sure the research questions are realistic and patient-centred. Institutional support, such as the registrar research support office, methodological and statistical help, and research tools, helps students plan, carry out, understand, and even publish their findings. All these supports are central for the successful completion of the research assignment.

The MMed research assignment is not just about enabling HPCSA specialist registration – it also encourages critical thinking and problem-solving and enhances the ability of students to interrogate the literature that will inform their clinical practice. Setting up simple structures where there are available research assignments to choose from allows students to choose projects that balance enthusiasm with feasibility, increasing the chance they will complete meaningful research within their training period. Departmental or divisional clarity on the disease burden treated locally, by means of basic ongoing data capture, enables a catalogue of potential relevant and viable research questions for the newly appointed registrar to choose from.

Although ground-breaking or novel research is not a requirement, studies that produce new insights could add to medical knowledge, especially if published. A publication leads to return of income to the institution with dual benefit in the form of a government subsidy. Therefore, it is beneficial for the student and the institution to answer clinically relevant research questions.^5^ This requires starting early by choosing an appropriate study design that is not overly ambitious, and by prioritising required approvals speeding up the project progress. In addition, encouraging internal collaboration by getting junior colleagues (students and allied health) involved in data collection where appropriate, promotes teamwork, practical experience, and even potential co-authorship if they contribute sufficiently. Accessible institutional support, methodological and statistical guidance within a coordinated structure could make the research process clearer, capitalising on its practical benefits. A positive first experience with research can motivate clinicians to continue engaging in evidence-based practice, helping them improve patient outcomes and strengthen the broader healthcare sector.

Within the South African context, healthcare resources are limited, and austerity budget cut measures are a reality. At the same time, we are dealing with a high number of patients with trauma and advanced disease (communicable and non-communicable), often within the constraints of operational hurdles. The MMed clinical research project is therefore a unique opportunity to provide answers to and develop skills to deal with these realities. Worldwide governing bodies are under pressure to deliver cost-effective, equitable health care, and South Africa is in a unique, albeit ‘forced’, position to provide answers on cost-effective yet safe patient management.^6,7^ Patient-centred research means that the patient’s experience is centrally placed in the research question, and the large numbers of patient populations with advanced disease place South Africa perfectly to provide answers relevant on the international stage.^8^

## Clinician as supervisor: Supervision and conflict management

Supervising MMed students involves guiding them in both clinical work and research, making it different from regular academic supervision. Unlike typical research degrees, MMed supervision goes beyond standard academic guidance, requiring supervisors to balance the practical and time constraints of clinical training – something the clinician supervisor is perfectly placed to do. This dual responsibility means supervisors need to be adaptable, helping students choose relevant topics, understand research methods, or recognise the need to collaborate with appropriate co-supervisors, and ensure students’ studies connect with real-world clinical issues. Many MMed students are new to research, so supervisors are often required to adjust their guidance to match each student’s experience and background, helping them grow as both doctors and researchers.^9^

The clinician supervisor should be cognisant of the demands on their student of balancing research, clinical work, teaching of younger colleagues, and endeavours outside of work such as family and friends and healthy living. Master of Medicine students have heavy workloads and tight schedules, which make it harder to dedicate time to research. Supervisors need to be aware of these pressures and should be flexible in how they support each student’s journey. This approach, known as ‘supervision in context’, requires supervisors to shape their guidance to fit the student’s clinical duties and level of research understanding.

Conflict is best mitigated by clear effective communication from the beginning and following best practices as set out below; however, the clinician supervisor should anticipate and be comfortable with handling challenging situations. Master of Medicine supervision is not just about academic advice; it is also about mentoring students on how to use research to improve their clinical work. Supervisors encourage students to see how research can lead to better patient care and inform medical decisions, an important skill to master before departing from the academic environment and embarking on a future career. To support students effectively, supervisors need to provide a structured framework to help them handle the mix of clinical and research roles.^10^

For resolving students’ conflicts, especially those involving the supervision team, co-supervisors and/or students, internal resolution should be prioritised in the interest of the student’s supervision. Where necessary, a neutral third party or a department head may be required to mediate to find a resolution. Supervisor–student conflict may also occur. Management of these includes the setting of clear expectations of both student and supervisor’s roles and responsibilities, regular discussions, and timeous response to any concerns raised. Conflict resolution strategies such as reframing issues, negotiating, and finding common ground enable supervisors to navigate these situations productively. General guidelines for conflict management in supervision include identifying the root causes, listening without assumption, and adapting conflict resolution styles to suit specific situations and personalities. Encouraging a culture of open dialogue, respect, and trust is essential, as is training in communication and conflict resolution skills. Follow-up on resolutions helps prevent future issues and fosters a positive supervision environment where students and supervisors can focus on academic and professional growth in a supportive setting.^11^

## Overview of the best practices and institutional support

The MMed Research Curriculum for Family Medicine at Stellenbosch, including a structured teaching of the entire MMed programme, is housed in a Moodle-based online learning support platform (FMHSlearn) with 12-week-long modules. Additional face-to-face teaching is required during 1 to 2-day contact sessions three times per year. The research curriculum features a dedicated Applied Research Module, which is characterised by pragmatic theory application, a deconstructed and scaffolded design, regular feedback, and extensive resources. The governance and supervision model are supportive and collaborative, featuring co-supervision by consultant supervisors and experienced primary supervisors.

The curriculum is constructively sequenced over 4 years, with the first year comprising an evidence-based medicine module to strengthen critical appraisal and begin to explore potential research topics.

The second year introduces the research module during which students refine their research questions, learn proposal writing, and are assigned supervisors. By the end of the module, students are expected to have a proposal almost ready for submission to the university research ethics committee.

The third year involves data collection, analysis, and report writing. Students also critique journal articles and attend 4th-year registrars’ research presentations during the contact sessions. Year 4 culminates in research presentations and publication discussions.

Effective supervision requires thoughtful planning, emphasising the division’s role in setting a solid structure. Supervisors benefit from ongoing training, like ‘Teach the Teacher’ workshops, which improve their mentorship skills. Co-supervision is encouraged, as it provides diverse expertise and shares the workload, while support from the Head of Department builds a strong research culture.

Early engagement is crucial; students should select topics and supervisors within 3 months of initial registration. Regular check-ins keep students on track, and internal peer reviews help streamline protocol approvals. Support systems, including research assistants and accessible data sets, further enhance the quality of students’ work. Communication extends beyond email, with regular face-to-face meetings, participating in internal and institutional research days, and internal ‘graduation days’ to monitor progress and celebrate achievements.

Supervising these projects offers academic and personal benefits, allowing supervisors to publish and advance their careers while guiding students. This approach fosters skilled specialists and nurtures a collaborative, research-driven culture that enriches both students and faculty.

## Recommendations

### Implement a structured supervision framework

Establishing a formalised structure for MMed supervision, especially for specialists who did not previously complete an MMed, is crucial for fostering consistency and clarity. This framework should outline key supervisory responsibilities, integrate regular check-in points, and provide a clear progression pathway for clinical research projects. By defining expectations for both supervisors and students, the supervision process becomes more transparent and manageable, enhancing the overall experience. This approach aligns with the supervision frameworks used in South African social work and healthcare sectors, which emphasise structured and supportive supervision practices.^12^

### Develop comprehensive clinical supervision training

Introducing and standardising clinical research supervision training is essential. This training should focus on mentorship skills, research ethics, and conflict management. Empowering supervisors with these skills will enable them to effectively address the complex needs of students, creating a supportive environment that significantly improves research quality and accelerates the professional development of MMed students. In South Africa, similar initiatives in clinical supervision for psychology and nursing have shown positive outcomes in professional development and service delivery.^13^

## Conclusion

Clinical research in South Africa is pivotal in addressing health disparities and developing new treatments and medical interventions to enhance healthcare outcomes. High-quality MMed research has the potential to provide solutions tailored to local healthcare needs. Improving the quality of MMed research requires us to acknowledge the challenge faced by clinicians who have limited research training. This lack of training may hinder their ability to effectively mentor students in developing rigorous scientific projects. Recognising this issue highlights the need for specific initiatives, such as foundational research training for supervisors, to enhance research capacity. By doing so, we can ensure lasting improvements in the quality of MMed supervision and outcomes. Future research could explore the challenges of ensuring quality MMed research supervision, particularly among clinicians with limited research training, and evaluate the effectiveness of existing supervisory skills programmes in addressing these gaps.
